# Risk Factors of Domestic Violence in Iran

**DOI:** 10.1155/2014/352346

**Published:** 2014-03-25

**Authors:** M. Rasoulian, S. Habib, J. Bolhari, M. Hakim Shooshtari, M. Nojomi, Sh. Abedi

**Affiliations:** ^1^Mental Health Research Center, Tehran Institute of Psychiatry, Faculty of Behavioral Sciences and Mental Health, Iran University of Medical Sciences, Tehran, Iran; ^2^Department of Community Medicine, Iran University of Medical Sciences, Iran

## Abstract

*Objectives.* In this study, we have evaluated the lifetime and past-year prevalence of exposure to physical violence among married women in the city of Tehran and urban and rural areas of Hashtgerd. *Methods.* The target population were noninstitutionalized female citizens, aged 15 years or older, who have at least one history of marriage and who resided in the capital city of Tehran or Hashtgerd County from the summer of 2008 to fall of 2010. We used a multistage sampling method. Tehran's District Six, a central district in Tehran, was selected as a representative cluster of all municipal districts in Tehran. A total of fifty blocks were randomly selected from this district, from which 1,000 married women aged 15 years or older were interviewed using a cross-sectional design. Data was gathered face-to-face using a structured questionnaire. The lifetime prevalence, past-year prevalence, and related factors of domestic violence were measured. SPSS version 11.5 was used for the analyses. *Results.* Figures for lifetime prevalence and past-year prevalence were measured to be 38.7% and 6.6%, respectively. The independent effects of marital status and location and type of residency for women, along with education and smoking habits of their spouses, were statistically significant in multivariate logistic regression analysis. *Conclusion.* Domestic violence is a public health concern in Iran. Based on our findings, we propose that empowering women through education, and improving their ability to find employment and income, along with increasing public awareness of human rights issues through education could lower the prevalence of domestic violence.

## 1. Introduction

Intimate partner violence is a worldwide problem, present in all cultures and societies [1]. It is important to understand that anyone can be abused and the most common feature of situation conducive to violence is an imbalance of power and control. Neither those who experience domestic violence nor the partners who abuse them fall into distinct categories. They can be of any age, ethnicity, income level, or level of education. Identifying potential risk factors is essential to develop preventive strategies [2].

It should be noted that violence of men against women is an increasingly acknowledged public health concern [3] and that it is associated with increased use of health care [4].

In a German research to determine the prevalence of domestic violence (DV), 1,557 female patients who were receiving medical care in a university hospital in Berlin, Germany, were studied. They ranged in age from 18 to 60 years. 70% of these patients accepted to participate in the study. Out of the participants, 57% of the victims of at least one episode of DV in their lifetime after the age of 16 claimed health consequences. 52% of the victimized women, who reported health consequences, had received medical care in lifetime including 33% surgery, 24% emergency department, and 10% received clinical treatment [5].

A 2006 World Health Organization study of 24,000 women from 10 different countries with different cultures found that 15%–71% had experienced physical or sexual violence from their partners at some point in their lifetime [6]. Another study on women's health and domestic violence found a highly significant association between lifetime experiences of partner violence and self-reported poor health [7].

In a cross-sectional study of 768 English-speaking women aged 18–64 who presented to 2 emergency departments in Ontario, risk indicators and exposure to intimate partner violence were evaluated. Women at the greatest risk of injury from domestic violence included those suffering depression or somatic symptoms and those who had an alcoholic male partner or a male partner without a full-time job [8].

Iran's Census Bureau, which is the country's official government agency responsible for data gathering, has never conducted a study on domestic violence and has not allowed international organizations to do so either. However, in 2004, the Women's Center for Presidential Advisory, Iran's Interior Ministry, and The Ministry of Higher Education decided to undertake a project in all 28 provinces of the country, regarding domestic violence. Based on the study, 66% of married women in Iran are subject to some kind of domestic violence in the first year of their marriage, either by their husbands or by their in-laws [9]. The study shows a direct correlation between having a higher level of education and being employed for women and experiencing a lower level of domestic violence. The study also shows that the higher the number of children in a family is, the more likely the woman will experience domestic violence.

To determine the prevalence of domestic physical violence against women and its associated risk factors, a study was conducted on 1,000 married women from Sanandaj (a city located in western Iran) [10]. The subjects were 1,040 women selected through multistage cluster random sampling. From a healthcare administrative perspective, the city was divided into 16 urban health centers, so the number of subjects selected from each was proportional to the size of the population served by each center. 15% of the women under study had been assaulted by their husbands at least once in the year prior to the data collection period and 38% of them at some time during their marriage. Economic problems were found to be the most frequent cause of domestic quarrels in the study. There was a significant association between subjects' husbands' educational level and violence against their wives. Physical violence against housewives was significantly more frequent than against employed women. The husbands' jobs were also strongly associated with the level of violence. Women who had children, on average, experienced a lower level of domestic physical violence [10].

Another study has looked at children to evaluate the lifetime prevalence of exposure to physical violence among parents [11]. 22.8% of the 1,495 high school students from Tehran reported they had witnessed instances of domestic violence between their parents. The prevalence of exposure among the girls was twice that among the boys. The most frequent act of violence was beating the partner with bare hands and the perpetrators of the violent acts were predominantly the fathers. Exposure was long lasting, and in those with more than one exposure, the mean duration of exposure was 5.1 years.

Another research in Sari (a city located in north of Iran) studied a thousand women and reported that 73.5% of the women studied were subject to physical abuse [12]. They were randomly selected from a list of 49,330 registered women who visited health centers in Sari.

They reported remarkably high rates of emotional abuse (80.8%) that was always higher than other types of abuse. Marriage at a young age, unemployment, low levels of education, substance misuse, presence of a physical or psychological problem, and having more children were cited as risk factors for domestic violence. Our objective in this study is to determine the risk factors of domestic violence in a large population of women aged 15 years and older who live in Tehran, the capital of Iran.

## 2. Methods

### 2.1. Population, Setting, and Sample Size

The study was conducted at the capital of Iran (Tehran) and Hashtgerd. Hashtgerd is a county 68 kilometers away from Tehran. This county includes rural and urban areas. In this study we considered the urban area of Tehran and the urban and rural areas of Hashtgerd. Tehran and Hashtgerd county have 7.1 million and 45,332 populations, respectively, based on the 2006 census.

Tehran has 22 municipal districts based on the 2006 census. We used a multistage sampling method. The target population was urban and rural noninstitutionalized female citizens, aged 15 years or older, married or divorced, who resided in Tehran city or Hashtgerd county in the year 2009.

Tehran's District Six was selected for this study, as this district is conveniently located at the heart of Tehran and it is well representative of all municipal districts in Tehran. It had a population of 237,29 in 2009. In this district, we randomly selected three subdistricts (areas), areas 11, 16, and 18. Based on the population of each area, 20, 15, and 15 blocks were randomly selected form areas 11, 16, and 18, respectively. For each block, we considered 10 households in order to select eligible subjects.

Therefore, a total of fifty blocks were randomly selected from District Six. For each block, a team of female interviewers approached the index household, which was selected as the first house at the left corner of each block. Then, the interviewers continued the enumeration in neighboring houses till they finished collecting the target sample size for each block. If more than one household inhabited a building, one was randomly selected. The interviewers introduced themselves by presenting their identification cards and describing the aims of the study to the members of the household. Then the married women of the households, ensuring safety and confidentiality issues, were invited for interviews and completing the questionnaire. If a woman refused to participate in the study, the neighboring house was approached next.

In Hashtgerd county, we used the same sampling method for our urban sample. Blocks were selected as clusters, and then, using the same method as in Tehran, the index house was selected. For selecting our rural sample, we first obtained the number of rural areas. Hashtgerd has thirteen villages. We selected all villages for the study. Then, through a proportional random sampling procedure, we selected subjects in each village based on its population size. In each village, between 5 and 60 eligible women were chosen from a randomly selected district. The index house was the first house on the bottom left corner of each district. The interviewers continued the enumeration in neighboring houses to collect the target sample size for that district.

In making a decision on the appropriate sample size, our goal was to determine the prevalence of domestic violence in Tehran and an area with both rural and urban populations. Given a proportion of 30% prevalence of physical intimate partner violence based on the literature, for a 95% confidence interval and precision of 0.032, and considering a design effect of 1.2, a target sample size of 1000 was determined. Half of the sample was collected from District Six of Tehran, and the other was collected from rural and urban areas of Hashtgerd. Therefore, from summer of 2008 to fall of 2010, 1000 married women aged 15 or older were interviewed in the capital city of Tehran and Hashtgerd county. The design of study was cross-sectional. The study carefully used female interviewers who were trained by expert psychologists and psychiatrists. The proposal for the study was approved by the institutional review board of the Faculty of Medicine, Iran University of Medical Sciences.

It should be noted that women with such experiences might be reluctant to acknowledge them due to various reasons such as shame or embarrassment. A small proportion of women think that participating in a study like this and providing information is equivalent to revealing “family secrets.”

### 2.2. Measures and Statistical Analysis

In this study, we used a structured questionnaire based on our objectives. This questionnaire was initially developed by the core research team of the protocol through a wide literature review. In order to achieve good content validity, the research team conducted an extensive review of the relevant literature. All items associated with physical intimate partner violence were extracted. Next, the initially developed questionnaire was reviewed and revised by a panel of experts. In order to evaluate its comprehensibility and readability, we pretested the final questionnaire on 20 women. The final version of the questionnaire included two parts and twenty-three items. Part 1 included fourteen questions about demographic variables related to women and seven questions about their partners. The second part included two main questions and a number of conditional follow-up questions for each. The first main question asked to the participating women was, “have you ever been intentionally hurt physically by your husband in your lifetime?” If the answer was positive, the women were then asked 13 follow-up questions about type and severity of the physical violence. The second main question in Part 2 asked about experiencing violence “during last year.” If a respondent answered positively to this question, she was asked 12 questions on the type and severity of the physical violence she had experienced.

Demographic variables measured in this study included age, education (“elementary,” “high school graduate,” or “college degree”), employment status (housekeeper, employed, or other), current marital status (married, single, or widow), duration of current marriage, socioeconomic status (rent/own the house, number of rooms per family member, and whether they have an automobile), number of living children, history of physical or mental disorders, drug use and smoking tobacco, and whether they had handicapped and physically or mentally ill children. There were also seven questions about the age, education, employment status, history of physical or mental disorders, and smoking and drug use status of the respondents' husbands. History of physical or mental disorder was defined as any physical or mental disorder that is chronic and needs persistent medication use or supervision of a physician. Smoking tobacco or drug use was measured as chronic cigarette smoking or regular consumption of any drugs such as heroin and morphine during their lifetime. We categorized all these materials as “substance use” in our analysis. In order to measure socioeconomic status of the respondents, we used home ownership status (own or rent), number of rooms per family member, and whether they had a car, because we assumed these items could reflect the socioeconomic status of women given the living style and standards of the society under study.

SPSS version 11.5 was used for the analyses. We measured means and standard deviations of the continuous variables and proportions of the discrete variables. For bivariate analysis of the association between physical domestic violence and demographic variables, we used independent sample *t*-tests, chi-square tests, and one-way analysis of variance (ANOVA). To find the adjusted association between domestic violence and risk factors, multiple logistic regression analysis was used. We included variables that had a statistically significant association with domestic violence according to our bivariate analysis into our logistic model. The threshold to determine statistical significance was set to 0.05.

## 3. Results

### 3.1. Demographic Characteristics

The data for this study were collected from a sample of 1,000 women from urban and rural areas of Tehran city and Hashtgerd county, Iran. Mean age of the respondents was 43.4 (13.2) and 3.4 percent of them had married twice or more. Mean age of their spouses was 49.1 (14.4) and 5.2% of the spouses had married twice or more in their lifetime. About one-third of the women (28.7%) had completed only the elementary school. About 80.5% of the women were housewives. Illness or disability was reported by 22.6% of the respondents. More than fifty percent of women's spouses had college or higher education. About four percent of the respondents' husbands were unemployed, and 27.4% of them were smokers. Other summary statistics of demographic characteristics of participants are illustrated in Table 1 based on location and type of residency. The difference between education and occupation levels of both women and their husbands across areas was statistically significant (*P* < 0.05). In Tehran, there were more women and spouses with higher education, and the proportion of retired spouses or spouses with clerical jobs was also higher in Tehran than Hashtgerd. Mental illnesses of women and their husbands were not significantly different between the three areas. There were more smokers among spouses in rural areas of Hashtgerd compared to the urban areas of this county, but the difference was not statistically significant.

### 3.2. Demographic Risk Factors for Domestic Violence

The lifetime prevalence of intimate partner violence (IPV) was 38.7%. The reported past-year prevalence of IPV was 6.6%. The lifetime prevalence of domestic violence was different across the three areas (*P* = 0.0001), but the difference in past-year prevalence across the areas was not significant (see Table 2).

Table 3 illustrates bivariate analysis of risk factors of IPV. Results show that domestic violence is reported more often in women with a lower level of education (*P* = 0.0001). Reported violence is more prevalent among housekeepers, but the difference is not statistically significant. Women with physical or mental illnesses were on average affected by domestic violence more than others (*P* < 0.05). Women's reports show that less educated husbands commit violence more than more educated ones. Spouses who had husbands with mental or physical illnesses are on average more prone to experience intimate partner violence (*P* < 0.05), and spouse's smoking is also found to be a risk factor for domestic violence (*P* = 0.0001).

### 3.3. Adjusted Risk Factors of Domestic Violence

Next we used a multivariate logistic regression model to further investigate the factors affecting domestic violence. As shown in Table 4, the independent effects of marital status, geographical location, education, and whether the spouse was a smoker remained statistically significant in this multivariate analysis. Divorced women are more likely to be a victim of domestic violence (OR = 5.46; 95% CI: [1.70 17.5]). Also women with a mental illness seem to be more prone to experience domestic violence than mentally normal women, although the *P* value is slightly higher than 0.05 (OR = 1.62; 95% CI: [0.94 2.79]). Residency in the urban areas of Hashtgerd has a significant independent effect on IPV (OR = 1.68; 95% CI: [1.13 1.50]). Women whose spouses only have an elementary school-level education experience a 1.59 times higher risk of experiencing domestic violence compared to women whose spouses have a college degree (95% CI: [1.0 2.54]). Women whose husbands have a history of mental illness seem to be more prone to be the victim of IPV with an odds ratio of 1.70 (95% CI: [0.92 3.12]), as the *P* value (0.08) is only slightly higher than our threshold. Smoking husbands are 6.5 times more likely to commit acts of domestic violence than nonsmokers (95% CI: [2.73 15.6]; *P* < 0.001).

## 4. Discussion

It is clear that violence of men against women is a public health concern with increased use of health care. Domestic violence results in prolonged stress, depression, anxiety disorders, posttraumatic stress disorders, suicide, eating disorders, and substance abuse [13]. In another study in Iran on 40 physically abused women that were referred to a local forensic medical center, the abused wives scored significantly higher on depression, anxiety, and stress, compared to a matched control group (DASS-21) [14].

In this study and among our random sample of 1,000 women, we observed that lifetime prevalence of exposure to physical violence was 38.7%, while 6.6% of the women had experienced physical violence in the year prior to the data collection period. It is interesting that the lifetime prevalence of domestic violence was different across the three geographical areas studied. Women living in urban areas of Hashtgerd county had the highest prevalence, while women in the capital city of Tehran had the lowest. We did not find a similar significant difference for the past-year prevalence.

The importance of socioeconomic status in the rate of IPV was confirmed in the present study. According to our findings, residents of urban areas of Hashtgerd were on average more likely to experience physical violence. Living in such areas is often associated with higher unemployment, lower education, and, in general, lower socioeconomic status. It can be interpreted as the women in urban areas of a new developed city like Hashtgerd have got higher knowledge about their rights, higher expression of such events, and more objections against IPV in comparison with rural areas of Hashtgerd. On the other hand, due to various reasons such as cultural standards and lack of financial independence, married women in rural areas are generally more fearful of the prospect of a separation, the difficulties of living as a divorced woman in a traditional society, or losing custody rights or even the right to visit their children following a divorce.

The lifetime prevalence of domestic violence was reported to be lower in a previous study in Tehran that estimated the occurrence of IPV in 22.8% of families [11]. That study was conducted on high school students, asking them about their parents in all 19 educational districts of Tehran in 2005. It is noteworthy that according to their findings, the socioeconomic status of the families and estrangement were significantly associated with the likelihood of exposure to domestic violence. It is possible that these students had not reported the true IPV due to emotional barriers such as fear and insecurity when disclosing a family secret.

Based on the findings of our study, domestic physical violence against currently divorced women was more prevalent (with an odds ratio of 5.46). It is reasonable to assume that they have had marital problems which resulted in getting a divorce. Wives with a lower level of education had a 1.59 times higher risk of being the victim of domestic violence compared to more educated women. It might be argued that educated people perhaps more effectively exercise coping and adaptation. One other potential explanation is that more educated women are better at anticipating problematic situations and trying to avoid them.

Another study of 400 married pregnant women in Kerman (a city located in the southeast of Iran) showed that other factors such as the husband's education, employment, and addiction status, along with geographical settings, are significantly correlated with the rate of IPV [15]. The strongest predictor of physical abuse in a multivariate regression model estimated in another research in Babol (a city in the north of Iran) was unemployment of the woman, whereas rural residence had a greater impact on psychological and sexual abuse [16].

A research on women in India found that some socioeconomic characteristics of women have a significant association with the occurrence of domestic violence. Urban residence, older age, lower education, and lower family income are associated with higher occurrence of domestic violence [17]. According to one other Indian study, age, education, occupation, marital duration, and husband's alcoholism emerged as significant predictors of victimization and perpetration of all types of domestic violence in India. They found a higher level of family income to be highly protective against the risk of violence [18].

In this study, we showed that women with a history of mental illness were more prone to be the victim of IPV (with an odds ratio of 1.70). Simple bivariate analysis provided support that having a husband with a mental illness is a significant risk factor for IPV. However, controlling for other variables, we did not find this variable to be a significant predictor of IPV in our multivariate logistic regression model.

Controlling for other variables, smoking husbands were found to be on average 6.5 times more likely to commit acts of physical violence against their wives (95% CI: [2.73 15.6]) compared to nonsmokers. The effect was also very statistically significant at *P* value smaller than 0.0001. Based on the size of effect of its statistical significance, smoking clearly is most strongly associated with the rate of IPV. One way of explaining this could be through a relationship between smoking and psychiatric disorder.

It appears that cultural factors have an important impact on the likelihood to experience domestic physical violence. Traditional societies have different attitudes toward women, women's roles, and their rights. A study of abused South African women found significant positive association between domestic violence and being victim of violence in childhood, having low levels of education, alcohol drinking habits of the women, and husband's boy child preference. No significant association was found with age of partners, employment, migrant status, financial disparity, household possessions, urbanization, marital status, and crowding [19].

Another study assessed Iranian women's opinion about domestic violence. It showed that 23% of women believed that their husbands had a right to commit violence against them [20].

In WHO multicountry study on domestic violence, the following item were asked of women: having sex with her partner in a number of situations, including if she is sick, if she does not want to have sex, if he is drunk, or if he mistreats her. In provinces of Bangladesh, Ethiopia, Peru, the United Republic of Tanzania, and Samoa, between 10% and 20% of women felt that women did not have the right to refuse sex under any of these circumstances [21]. While over three quarters of women in the urban settings of Brazil, Japan, Namibia, and Serbia and Montenegro said that no reason justified violence, at most only a quarter thought so in the provincial settings of Bangladesh, Ethiopia, Peru, and Samoa. In all the countries, female infidelity was the most widely accepted justification of violence, but the range was wide: from 80% in Ethiopia to 6% in Serbia and Montenegro. Disobeying a husband was the next most widely accepted reason [21]. It should be noted that according to this study, acceptance of wife beating was higher among women who had experienced abuse. It may show that women sometimes learn to “accept” violence in situations where they are victims.

In Arab and Islamic countries such as Egypt, Palestine, and Tunisia, studies showed that at least one out of every three women is under physical violence by her husband [6, 22]. However, it is possibly a result of culture rather than religion [23].

A study in Egypt found that the 12-month prevalence of wife beating was lower only when both partners were educated, and more educated women experienced less severe forms of wife beating than less educated women [24].

Domestic violence is most strongly related to the status of women in a society and to the normative use of violence in conflict situations or as part of power struggles. Living in a culture, which considers males as dominant figures and females as second-class citizens, predisposes female as being a victim.

It appears that false beliefs, low educational level of women, lack of knowledge about their rights, and low social support for abused women play an important role in continuation of domestic violence.

## 5. Conclusion

In this research, we performed a large-scale field study of 1,000 Iranian women in three distinct geographical areas. Our findings show that empowering women through education and helping to improve their ability to find employment and a steady income could help significantly reduce domestic violence. Also encouraging men to allow their wives to be more actively involved in decision making is another important step that could help reduce domestic violence against women. Increasing public awareness of human rights through education and outreach programs is a very important measure that should be undertaken through reliable local resources within communities.

## Figures and Tables

**Table 1:** Summary statistics of demographic characteristics of the studied women grouped based on location and type of residency (*n* = 1000).

Variable	Hashtgerd (urban) (*n* = 252)	Hashtgerd (rural) (*n* = 247)	Tehran (*n* = 501)	*P* value
Education				0.0001
Elementary	120 (47.6)	107 (43.3)	60 (12.2)	
High school graduate	51 (20.2)	71 (28.7)	72 (14.4)	
College	81 (32.1)	69 (27.9)	369 (73.7)	
Occupation				0.0001
Housekeeper	232 (92.1)	238 (96.4)	335 (66.9)	
Employed	12 (4.8)	5 (2.0)	99 (19.8)	
Other	8 (3.2)	4 (1.6)	67 (13.4)	
Current marital status				0.02
Married	226 (89.7)	237 (96.0)	466 (93.0)	
Divorced	5 (2.0)	5 (2.0)	12 (2.4)	
Widow	21 (8.3)	5 (2.0)	23 (4.6)	
Home ownership				0.0001
Owned	164 (65.1)	205 (83.0)	319 (63.7)	
Rented	68 (27.0)	34 (13.8)	173 (34.5)	
Other	20 (7.9)	8 (3.2)	9 (1.8)	
Physical illness				0.20
Yes	67 (26.6)	51 (20.6)	108 (21.6)	
No	185 (73.4)	196 (79.4)	393 (78.4)	
Mental illness				0.66
Yes	21 (8.3)	17 (6.9)	33 (6.6)	
No	231 (91.7)	230 (93.1)	468 (93.4)	
Handicapped children				0.60
Yes	10 (4.0)	8 (3.2)	14 (2.8)	
No	242 (96.0)	239 (96.8)	486 (97.2)	

Spouse's demographic information				
Education				0.0001
Elementary	104 (41.4)	85 (34.4)	41 (8.2)	
High school graduated	54 (21.5)	85 (34.4)	75 (15.1)	
Academic	93 (37.1)	77 (31.2)	382 (76.7)	
Occupation				0.0001
Unemployed	9 (3.6)	14 (5.7)	20 (4.0)	
Worker/farmer	146 (58.2)	157 (63.6)	177 (35.5)	
Clerk/retired	71 (28.3)	65 (26.3)	273 (54.8)	
Other	25 (10.0)	11 (4.5)	28 (5.6)	
Physical illness				0.01
Yes	56 (22.3)	41 (16.6)	70 (14.1)	
No	195 (77.7)	206 (83.4)	428 (85.9)	
Mental illness				0.40
Yes	16 (6.4)	10 (4.0)	29 (5.8)	
No	235 (93.6)	237 (96.0)	469 (94.2)	
Smoking				0.30
Yes	67 (26.7)	73 (29.5)	134 (26.9)	
No	184 (73.3)	174 (70.4)	364 (73.1)	

**Table 2:** Lifetime prevalence and past-year prevalence of domestic violence by location and type of residency.

Domestic violence	Hashtgerd (urban)	Hashtgerd (rural)	Tehran	*P* value
Lifetime prevalence				0.0001
Yes	124 (49.8)	92 (37.4)	171 (34.1)	
No	125 (50.20)	154 (62.6)	330 (65.9)	
Past-year prevalence				0.12
Yes	23 (9.3)	12 (4.9)	31 (6.2)	
No	225 (90.7)	234 (95.10)	470 (93.8)	

**Table 3:** Lifetime prevalence of domestic violence based on women's characteristics.

Characteristic	No domestic violence (*n* = 609)	Domestic violence (*n* = 387)	*P* value
Education			0.0001
Elementary	147 (24.1)	139 (35.9)	
High school graduate	115 (18.9)	78 (20.2)	
College	347 (57.0)	170 (43.9)	
Home ownership			0.39
Owned	427 (70.1)	258 (66.7)	
Rented	163 (26.8)	112 (28.9)	
Other	19 (3.1)	17 (4.4)	
Occupation			0.15
Housekeeper	478 (75.8)	323 (83.5)	
Employed	77 (12.6)	39 (10.1)	
Other	54 (8.8)	25 (6.4)	
Current marital status			0.0001
Married	583 (95.7)	342 (88.4)	
Divorced	4 (0.7)	18 (4.7)	
Widow	22 (3.6)	27 (7.0)	
Physical illness			0.02
Yes	124 (20.4)	102 (26.4)	
No	485 (79.6)	285 (73.6)	
Mental illness			0.002
Yes	31 (5.1)	40 (10.3)	
No	578 (94.9)	347 (89.7)	
Handicapped children			0.14
Yes	15 (2.5)	16 (4.1)	
No	593 (97.5)	371 (95.9)	

Spouse's demographic characteristics			
Education			0.0001
Elementary	112 (18.5)	118 (30.6)	
High school graduate	123 (20.3)	91 (23.6)	
College	371 (61.2)	177 (45.9)	
Occupation			0.65
Unemployed	24 (4.0)	19 (4.9)	
Worker/farmer	286 (47.2)	190 (49.2)	
Clerk/retired	261 (43.1)	150 (38.9)	
Other	35 (5.8)	27 (7.0)	
Physical illness			0.02
Yes	89 (14.7)	78 (20.2)	
No	517 (85.3)	308 (79.8)	
Mental illness			0.001
Yes	22 (3.6)	33 (8.5)	
No	584 (96.4)	353 (91.5)	
Smoking			0.0001
Yes	141 (23.3)	131 (33.9)	
No	465 (76.7)	255 (66.1)	

**Table 4:** Logistic regression analysis of risk factors of lifetime prevalence of domestic violence.

Variable	Coefficient estimate	*P* value	Odds ratio	CI for OR
Characteristics of wives				
College education			Reference	
High school graduate	0.056	0.81	1.05	0.66–1.68
Elementary	0.18	0.35	1.20	0.81–1.79
Married women			Reference	
Divorced	1.69	0.004	5.46	1.70–17.5
Widow	0.26	0.44	1.30	0.65–2.59
Mental illness	0.48	0.07	1.62	0.94–2.79
Physical illness	0.07	0.67	0.92	0.64–1.33
Handicapped children	0.30	0.44	1.35	0.62–2.94
Tehran			Reference	
Hashtgerd rural	0.004	0.98	1.00	0.67–1.50
Hashtgerd urban	0.51	0.009	1.68	1.13–1.50

Spouse's characteristics				
Age	0.006	0.30	1.00	0.99–1.02
College education			Reference	
High school graduate	0.26	0.17	1.30	0.88–1.92
Elementary	0.46	0.05	1.59	1.0–2.54
Physical illness	0.04	0.82	1.04	0.70–1.55
Mental illness	0.53	0.08	1.70	0.92–3.12
Smoking	1.87	0.0001	6.50	2.73–15.6
